# Treatment of Purpura Hemorrhagica with Antistreptococcus Serum

**Published:** 1898-01

**Authors:** W. G. Hollingsworth

**Affiliations:** Utica, N. Y.


### TREATMENT OF PURPURA HEMORRHAGICA WITH ANTISTREPTOCOCCUS SERUM.

By W. G. Hollingsworth, D.V.S.,
UTICA, N. Y.

*Subject:* Bay gelding. *History:* Convalescent from pneumonia, about ready for light work. Hygienic conditions of stable very poor.

*Symptoms.* First observed some small swellings on the extremities, which rapidly attained an unusual size, the peculiarly characteristic symptoms made diagnosis easy. The swellings, after a day or two, suddenly disappeared, and the animal evinced much uneasiness and pain, which was followed by loose, bloody discharges from the bowels. This condition was followed later by the temperature rising to 105° F.; pulse, 96; respirations, 36.

*Treatment.* The usual treatment was prescribed at the onset, but I determined to try Pasteur's antistreptococcus serum, and at once ordered some from their Chicago representative. On its arrival I at once proceeded to administer the same, as the condition of the horse was much worse, the temperature, respirations, and pulse all accelerated, pain marked, and flux of the bowel. To relieve the pain a hypodermic injection of morphine was given first, after which 10 c.c. of the serum was introduced, with instructions to repeat the latter dose every three hours until my return, with cannabis Indica as a soporific when in pain. These instructions were carried out by a very careful and intelligent attendant, and on my return, at 5 P.M. of the same day, I found the animal much easier. The same treatment was followed through the night, and at my morning call the next day the temperature had dropped to 103° F., pulse 50, repirations 20, and patient disposed to pick hay, but refused a bran-mash offered him. Ordered the 10 c.c. doses of serum every six hours and returned at night, when the temperature had receded to 100° F., pulse 50, and respirations 16, and anxious to eat; discharges from bowels better in appearance; no hemorrhage, and fecal matter tending to ball. The improvement was marked, and the frequency of the injection was gradually reduced until but one a day was given. The swellings after some days threatened to return, and the dose of serum was given every eight hours and the swellings gradually disappeared. The dose was reduced to two a day, then to one a day, for four days, after which I placed him on tonics. He had thirty-three doses of 10 c.c. each of the serum. Marked improvement followed the serum, so I decided to give it more extended trial, and have placed it in stock for future emergencies.

